# Short-term microgravity effects simulation does not affect fNIRS measures of cerebral oxygenation changes induced by cognitive load

**DOI:** 10.3389/fphys.2025.1425302

**Published:** 2025-02-19

**Authors:** Vsevolod Peysakhovich, Thibault Kiehl, Lucia Vicente Martinez, Laure Boyer, Mickaël Causse, Alexis Paillet, Anne Pavy-Le Traon

**Affiliations:** ^1^ Fédération ENAC ISAE-SUPAERO ONERA, Toulouse, France; ^2^ Spaceship FR, CNES, Toulouse, France; ^3^ MEDES, Institute for Space Physiology and Medicine, Toulouse, France; ^4^ Neurology Department, University Hospital of Toulouse, Toulouse, France

**Keywords:** fNIRS, cognitive load, cerebral oxygenation, head-down tilt, n-back

## Abstract

In the past decade, there has been a surge in interest in space exploration studies, particularly due to the prospect of exploring distant planets such as Mars. However, long-duration space missions may pose cognitive challenges resulting from spaceflight-induced perceptual and motor changes, prolonged cephalic fluid shifts, and high cognitive load. One method for monitoring cognitive activity is functional near-infrared spectroscopy (fNIRS), a technique not yet tested under prolonged microgravity conditions beyond parabolic flight periods. Since fNIRS relies on cerebral oxygenation levels, should we adjust it for the fluid shift? To address this, the study explores the impact of simulated microgravity on cerebral oxygenation measures using fNIRS during a cognitive task, employing head-down tilt at different inclination levels and the Toulouse N-back Task (assessing memory and mental calculation) with varying difficulty levels. Eighteen subjects participated in the experiment. The results indicated that increasing difficulty levels of the cognitive task led to decreased accuracy, longer response times, and higher perceived difficulty scores. The inclination levels did not affect task performance. Increased difficulty was also concomitant with increasing HbO and decreasing HbR concentrations unaffected by the head-down tilt angle variations. These promising findings suggest that fNIRS measures could be used under microgravity conditions to measure cognitive load without correction for fluid shift.

## 1 Introduction

During the last decade, humanity witnessed an increased interest in space exploration accompanied by the democratization of access to and beyond Karman’s line: a new space station assembly, docking of a module to the International Space Station, increasing commercial spaceflight, development of new crewed vehicles, Starship tests, etc. In a few years, professional astronauts may walk on the Moon and undertake a voyage to Mars, while undertrained space tourists rush on the low orbit with short hops, in-orbit sojourns, or even extra-vehicular walks. However, humans are not designed to function in space and many aspects relative to cognitive functioning in this environment remain challenging. Spaceflight is known to induce perceptual and motor deficits (Clément and Reschke, 2010; Kuldavletova et al., 2023; Tays et al., 2021), and cognitive load is an important factor that can further affect motor performance (Burcal et al., 2019) and be increased by vestibular deficits (Clément et al., 2023; Bigelow and Agrawal, 2015; Hanes and McCollum, 2006). Although the literature does not suggest clear evidence of the deleterious impact of spaceflight on cognitive performance (Strangman et al., 2014; Casler and Cook, 1999), exposure to radiation during deep space missions could negatively impact cognitive function (Cacao and Cucinotta, 2019; Rabin et al., 2014; Acharya et al., 2019). Hence, it is crucial to monitor the cognitive effort of astronauts both to continue sustaining the high-level cognitive performance of current operations and prevent possible deficits associated with deep space exploration.

Cognitive load can be estimated using self-report measures (Naismith et al., 2015), objective monitoring of the body activity (heart rate, respiration rate, eye movements, brain activity) (Ayres et al., 2021), secondary-task performance (Park and Brünken, 2015; Greenberg and Zheng, 2022), or observer ratings. Most of these measures present disadvantages for online monitoring: self-report and observer ratings are subjective and might not reflect the true cognitive load; the secondary-task method interrupts the operator and is not suitable in operational settings; cardiac and respiratory measures are more sensitive to the stress and arousal rather than workload; eye movements might reflect the cognitive load but mostly reveal the visual attention distribution. Brain activity measurement techniques such as electroencephalography (EEG, Chikhi et al., 2022; Ghani et al., 2020; Antonenko et al., 2010) or functional near-infrared spectroscopy (fNIRS, Ferrari and Quaresima, 2012; Aghajani et al., 2017; Herff et al., 2014) are good candidates for measuring cognitive load.

EEG is a technique that was already successfully used for scientific experiments aboard the International Space Station for many years (Fiedler et al., 2023; Van Ombergen et al., 2017). fNIRS measurement is a more recent technique that has been successfully used to measure cognitive load in numerous studies, consistently demonstrating increased activity in prefrontal regions as task difficulty rises (Causse et al., 2017; Ayaz et al., 2012; Mandrick et al., 2016). This increase reflects heightened activation in areas responsible for critical cognitive processes, such as rational reasoning (Donoso et al., 2014), working memory (Funahashi, 2017), planning (Tanji and Hoshi, 2001), and more. During effortful tasks, the increased demands on executive function are reflected by heightened prefrontal cortex activity (Shenhav et al., 2017), a region shown to exhibit a linear increase in activity with working memory (WM) load (Braver et al., 1997). These studies collectively suggest that the prefrontal cortex region, in particular, dorsolateral ones can serve as a reliable proxy measure of mental workload.

Generally, fNIRS has a good signal-to-noise ratio and is less sensitive to electrical noise compared to EEG (Hasan et al., 2020; Ghafoor et al., 2021), as it measures hemodynamic changes (oxygenated/deoxygenated hemoglobin) in response to brain activity. EEG can be affected by artifacts from muscles, movement, breathing, or external electrical interference, including radio communications. However, advanced filtering methods are available for EEG, and the setup of both techniques involves similar constraints in terms of electrode placement and comfort, especially considering the recent generalization of dry EEG electrodes.

fNIRS has already been tested in microgravity during parabolic flights (Zhang et al., 2011; Schneider et al., 2013) or real flight of light aircraft (Dehais et al., 2018; Gateau et al., 2018). However, compared to only a few seconds of short microgravity periods or short aircraft tests, longer stays in microgravity associated with human spaceflight could be very challenging for fNIRS measurements. fNIRS is an optical imaging method that detects changes induced by brain activity in oxygenated (oxyHb) and deoxygenated (deoxyHb) hemoglobin blood levels (León-Carrión and León-Domínguez, 2012). In microgravity, the redistribution of fluid towards the brain also alters the cerebral hemodynamics (Kawai et al., 2003; Du et al., 2021; Lee et al., 2021). Hence, to monitor online cognitive load using fNIRS, we need to understand whether the measurements of cognitive load levels by this technique are affected by microgravity-induced fluid shift. In particular, we used head-down tilt simulated microgravity with different inclination levels (pre-0°, −10°, −20°, post-0°) and a cognitive task with multiple levels of difficulty (Toulouse N-back Task with n = 0, 1, and 2). While the tilted participants performed the task, their prefrontal cortex oxygenation levels were monitored using a simple two-channel fNIRS system. An increase in task difficulty should result in an increase in blood oxygenation in the prefrontal area, regardless of the level of tilt, provided that tilt will not affect the measurements.

## 2 Materials and methods

### 2.1 Subjects

Eighteen healthy volunteers, 9 men and 9 women (age 24.28 ± 3.56 years, height 173.28 ± 9.39 cm, weight 67.56 ± 9.59 kg), were enrolled in the study. They were all in good physical and mental health, and were naive to head-down tilt experiences They had no history or clinically relevant signs of cardiovascular, neurological, or ear-nose-throat pathology, no objective signs of venous insufficiency, and all were non-smokers. All volunteers had normal or corrected-to-normal (using lenses) vision.

The protocol was conducted following the Declaration of Helsinki and approved by the Ethics Committee (CPP 23.01719.000252) and the French Health Authorities. The study was carried out and promoted by the Institute for Space Medicine and Physiology (MEDES, Toulouse France) and sponsored by the French Space Agency (CNES, Spaceship FR project).

All volunteers signed a written consent form before the experiment and were aware of their right to withdraw from the experiment without prejudice at any time. The volunteers performed two experimental sessions: one described in the present study; and another one with a different set of sensors used by another research team and beyond the scope of the present paper.

### 2.2 Head-down tilt setup

Head-down tilt (HDT) is a relevant experimental setup for simulating the effects of microgravity on the human body (Watenpaugh, 2016), particularly fluid-shift (London et al., 1983; Watenpaugh, 2016). During the experiment, the volunteers were placed in a supine position on a bed that could be tilted to different angles according to head-to-toe axis. It allows the head to be lower than the lower extremities and induces fluid shift. Other axes such as lateral (lying on one side) or dorsoventral (lying face up vs. down) could have slight effects on local tissue perfusion, but these changes are much smaller in scale compared to fluid shifts along the head-to-toe axis.

The researchers often use multiples of 6° (Marshall-Goebel et al., 2017) or multiples 10° (Kermorgant et al., 2022; Cooke et al., 2003) as possible tilt angle values. We chose to use multiples of 10° to maximize possible differences between conditions. The tilt angle was verified using both analog and digital inclinometer. The cognitive task was displayed on a laptop computer screen placed above the participants’ chests mounted on a hospital table with adjustable height and inclination. The responses were given using a CedrusBox response box placed on the participants’ abdomen at a comfortable length reachable by both hands. The response box had two buttons: green for “yes” and red for “no”.

### 2.3 Experimental design

The participants were head-down tilted for 20 min at each position at 0°, −10°, −20°, and back to 0°. This protocol enabled a controlled and progressive fluid shift from 0° to −20°. However, returning to a baseline position [e.g., seated posture, as in Chouchou et al. (2020)] was not feasible due to time constraints. Such a transition would have extended the protocol by over an hour without offering substantial scientific value. Existing research indicates that a resting tilt duration of approximately 6 min is sufficient to elicit a cardiovascular hemodynamic response (Whittle et al., 2022), while around 15 min is enough to induce cerebral hemodynamic changes (Kato et al., 2024).

**FIGURE 1:**
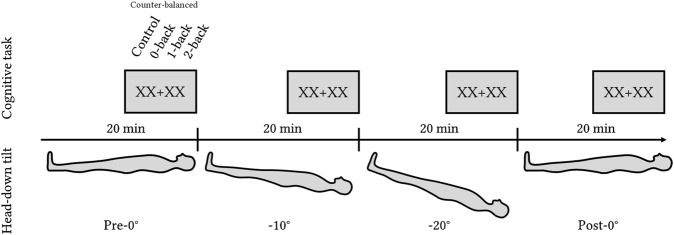
Experimental design with four levels of HDT angles: pre-0°, −10°, −20°, and post-0°. Each position lasted for 20 min with the cognitive task (Toulouse N-back Task) administered during the last 5 min of each level.

In the analysis, we refer to it as the HDT angle factor with four levels: Pre-0°, −10°, −20°, and Post-0°. The total experimental time was 80 min. After 15 min of tilt in a given position, volunteers completed the cognitive task (Toulouse N-back Task), followed by the NASA-TLX and Perceived n-back difficulty questionnaires. Figure 1 shows the order of the tilt levels.

### 2.4 Toulouse N-back task

At the end of each tilt, the participants performed the cognitive task called the Toulouse N-back Task (TNT, Mandrick et al., 2013; Causse et al., 2017). TNT is an adaptation of a classical n-back task where the participants perform the n-back task on the results of arithmetical operations. The arithmetic operations consisted of addition/subtraction of multiples of 10 between 10 and 90 (for example, 10 + 40 or 90–30).

**FIGURE 2:**
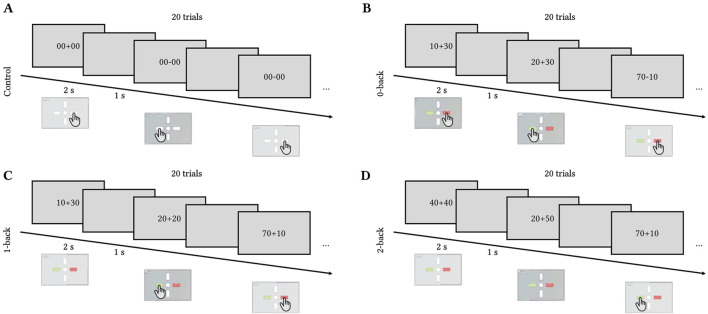
Toulouse N-back Task timecourse. Each trial lasted for 2 s with 1 s inter-trial interval. Each block of difficulty included 20 trials. **(A)** Control (press left or right button), **(B)** 0-back (compare the result to 50), **(C)** 1-back (compare the result to the previous one), **(D)** 2-back (compare the result to the one obtained two trials ago).

The task had three levels of difficulty (0-, 1-, and 2-back) and a control reaction time task to control for motor response where the participants had to press any button as soon as the stimulus (either “00 + 00″ or “00–00″) appeared on the screen. In the 0-back condition, the participants had to compare the result of the operation with 50. In the 1-back condition, the participants had to compare the result of the operation with the result of the previous one. In the 2-back, they had to compare the results with the result obtained two operations before. Figure 2 illustrates the four conditions.

The three difficulty levels were counterbalanced across tilt levels and participants. Each trial lasted for 2 s with 1 s of inter-stimulus interval. Each block contained 20 trials ((2 + 1) × 20 = 60 s per level) preceded by a 10-s countdown notifying the participants of the upcoming difficulty level. The total duration of the cognitive task per tilt level was 280 s (4 min 40 s). The participants underwent 15 min of training on the Toulouse N-back Task at the end of the inclusion visit if met all inclusion criteria.

### 2.5 Data acquisition

During the cognitive tasks, the response times and accuracy were recorded using a Cedrus response box. At the end of each tilt condition, once the cognitive task was completed, the participants filled out the NASA-TLX questionnaire evaluating the mental, physical, and temporal demands, performance, effort, and frustration levels associated with a given tilt. After NASA-TLX completion, the participants were asked to rate the difficulty of each n-back level (0-, 1-, or 2-back) on a scale from 1 (very easy) to 7 (very hard).

The hemodynamic responses (fNIRS signal) were recorded using the low-cost fNIRS device by biosignalplux (PLUX, Lisbon, Portugal). The fNIRS device was equipped with two sensors comprising each an infrared emitter with a peak at 860 nm, a red emitter with a peak at 660 nm, and a detector placed at 20 mm. fNIRS measures are limited to the outer cortex of the brain, roughly 5–8 mm of the brain's surface (Santosa et al., 2018), and cannot record activity in the subcortical area. The fNIRS data were recorded at 10 Hz and a resolution of 8 bits. Two sensors were maintained on participants’ foreheads using a flexible headband roughly at the Fp1 and Fp2 positions of the international 10/20 placement system, corresponding to the dorsolateral prefrontal cortex region. The fNIRS data was transmitted to the computer via Bluetooth using OpenSignals software, sent to Lab Streaming Layer, and recorded using Lab Recorder software.

### 2.6 Data processing

#### 2.6.1 Behavior

For each participant and each Toulouse N-back Task session, we computed the number of correct responses and the correct response times (in s ). The response accuracy was computed as the ratio of correct responses ranging from 0 to 1 and weighted according to the n-back level (20 possible correct responses for the 0-back, 19 for the 1-back, and 18 for the 2-back). The correct response times were averaged for each difficulty level.

#### 2.6.2 fNIRS

First, we converted the raw intensities for each wavelength to optical density using the formula $OD=-log\left(\frac{I}{I_{0}}\right)$ , where OD is the optical density, I is the raw light intensity, and $I_{0}$ is the reference intensity, where we used the average value of the whole intensity recording. Then, we applied the Modified Beer-Lambert law (MBLL) and obtained HbO and HbR concentrations from inverting the extinction coefficient matrix using MATLAB inv function. We used a fixed differential pathlength factor (DPF) value of 6.06 for all participants and molar extinction coefficients corresponding to the 660 nm and 860 nm wavelength (Wray et al., 1988).

The data was filtered using a moving average filter with a window size of 50 samples (Pinti et al., 2019). The HbO and HbR signals were then averaged over 60-second periods for each task level (control, 0-, 1-, and 2-back) for each HDT angle value. As a baseline procedure, we used subtraction of the averaged HbO and HbR signals during the 10 s preceding the cognitive task at each HDT level. The data were averaged between two channels. Both channels were excessively noisy for five participants, and these participants were removed from the HbO and HbR analyses. All data processing was performed using MATLAB custom-made scripts.

#### 2.6.3 Statistical analyses

Statistical analyses were carried out using JASP software. We performed repeated measures analyses of variance (rm-ANOVAs) for accuracy, response times, task load index, perceived n-back difficulty, and HbO and HbR concentration changes. The factor levels are explicited in the following section for each result. Greenhouse-Geisser correction was applied for p-values when necessary.

## 3 Results

### 3.1 Behavior

#### 3.1.1 Accuracy

**FIGURE 3:**
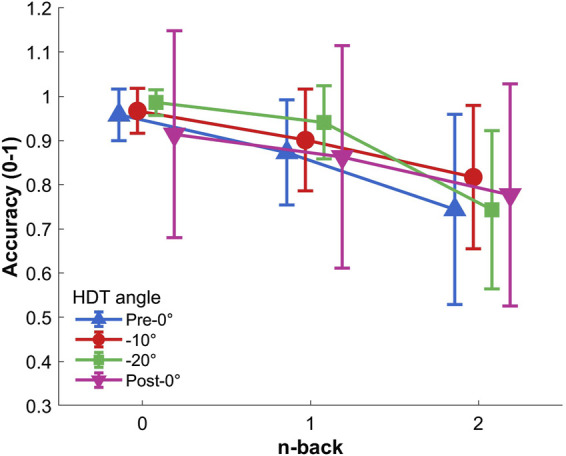
Response accuracy to the Toulouse N-back Task according to each level of difficulty and HDT angle. Vertical bars represent standard deviation.

Two-way repeated measures ANOVA (0-back, 1-back, and 2-back × Pre-0°, −10°, −20°, and Post-0°) showed a significant effect of the n-back level on the accuracy, $F\left(2,34\right)=23.816,p<.001,\eta_{p}^{2}=.58$ (Figure 3). The post-doc analysis showed that the participants’ accuracy during the 2-back condition was lower compared to both the 0-back and 1-back conditions (p<.001) , and 1-back accuracy was lower than 0-back accuracy (p=.032) . Nor HDT angle effect, $F\left(3,51\right)\;=\;0.640,p=.474,\eta_{p}^{2}=.04$ , nor the interaction of the factors, $F\left(6,102\right)\;=\;1.894,p\;=.122,\eta_{p}^{2}=.10$ , were significant.

#### 3.1.2 Response times

##### 3.1.2.1 Control reaction task included

**FIGURE 4:**
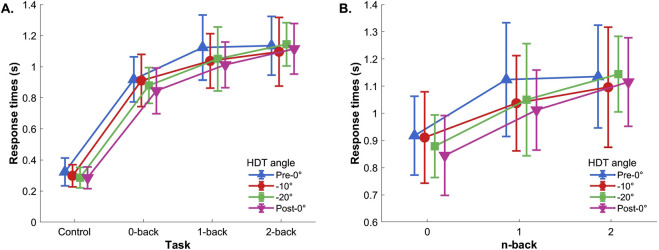
Reaction time. **(A)** With control task and **(B)** without control task. Vertical bars represent standard deviation.

Two-way repeated measures ANOVA (Control, 0-back, 1-back, 2-back × Pre-0°, −10°, −20°, and Post-0°) showed a significant effect of the task (control, 0-, 1-, and 2-back), $F\left(3,42\right)=143.726,p<.001,\eta_{p}^{2}=0.91$ , and significant effect of the tilt angle, $F\left(3,42\right)=4.259,p=.017,\eta_{p}^{2}=0.23$ . No significant interaction was found, $F\left(9,126\right)=1.009,p=.414,\eta_{p}^{2}=.067$ (Figure 4A). Post hoc comparisons showed that response times during the control task were lower compared to all n-back levels (all p<.001 ), and that response times during the 0-back condition were significantly lower compared to 1- and 2-back (both p<.001 ). Regarding the HDT angle effect, the *post hoc* analysis showed that response times were lower during post-0° tilt compared to pre-0° tilt (p=.006) .

##### 3.1.2.2 Control reaction task excluded

If the control task was excluded from the analysis, the two-way repeated-measures ANOVA (0-back, 1-back, and 2-back × Pre-0°, −10°, −20°, and Post-0°) showed a significant main effect of the n-back level, $F\left(2,32\right)=21.251,p<.001,\eta_{p}^{2}=0.57$ , and no effects of the HDT angle, $F\left(3,48\right)=2.489,p=.083,\eta_{p}^{2}=.135$ , nor interaction, $F\left(6,96\right)=1.250,p=.299,\eta_{p}^{2}=.072$ (Figure 4B). The *post hoc* showed that the response times during 0-back were significantly lower compared to 1- and 2-back (both p<.001 ).

#### 3.1.3 Subjective measures (task load index and perceived n-back difficulty)

**FIGURE 5:**
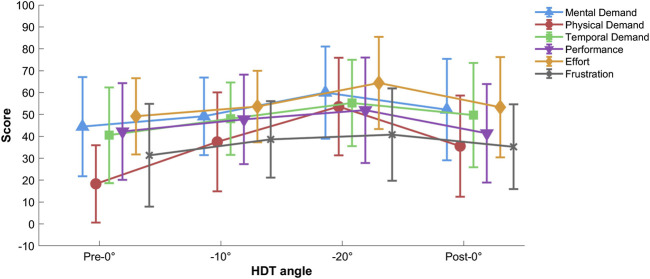
Task load index per HDT angle. Vertical bars represent standard deviation.

The two-way repeated-measures ANOVA (Mental demand, Physical demand, Temporal demand, Performance, Effort, Frustration × Pre-0°, −10°, −20°, and Post-0°) showed a significant main effect of Task Load Index dimension during the n-back task, $F\left(5,85\right)=7.842,p<.001,\eta_{p}^{2}=.316$ . The *post hoc* comparisons showed that physical demand was scored lower compared to mental and temporal demands, and to effort ( p=.003,p=.03 , and p<.001 respectively). Also, frustration was scored lower compared to temporal demand (p=.033) and effort (p<.001) . The ANOVA also revealed a significant effect of HDT angle, $F\left(3,51\right)=5.974,p=.009,\eta_{p}^{2}=.260$ , with *post hoc* analysis showing a higher overall task load index during −20° tilt compared to the pre-0° condition (p<.001) . Eventually, there was a significant interaction of these two factors, $F\left(15,255\right)=3.598,p=.002,\eta_{p}^{2}=.175$ (cf. Figure 5). The *post hoc* analysis showed that physical demand was scored lower compared to mental and temporal demand, performance, and effort (maximum p=.002 ) only for the pre-0°, see Figure 5. Additionally, during the −20° tilt condition, effort was scored higher compared to frustration (p<.001) .

**FIGURE 6:**
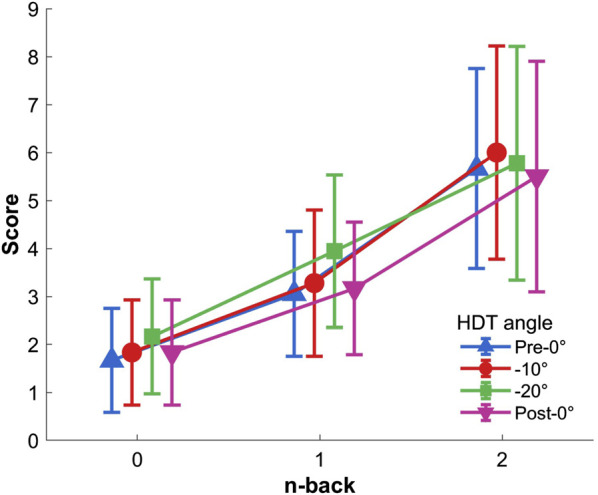
Perceived n-back difficulty per HDT angle. Vertical bars represent standard deviation.

The two-way repeated-measures ANOVA (0-back, 1-back, and 2-back × Pre-0°, −10°, −20°, and Post-0°) indicated a significant main effect of n-back level on perceived difficulty, $F\left(2,34\right)=82.909,p<.001,\eta_{p}^{2}=.830$ (Figure 6). Post hoc indicated that 0-back was scored significantly lower than both 1- and 2-back, and that 1-back was scored significantly lower compared to 2-back (all p<.001 ). No HDT angle effect, $F\left(3,51\right)=1.666,p=.196,\eta_{p}^{2}=.089$ , nor interaction, $F\left(6,102\right)=1.439,p=.237,\eta_{p}^{2}=.078$ , were found.

### 3.2 fNIRS measures

#### 3.2.1 HbO concentration changes

**FIGURE 7:**
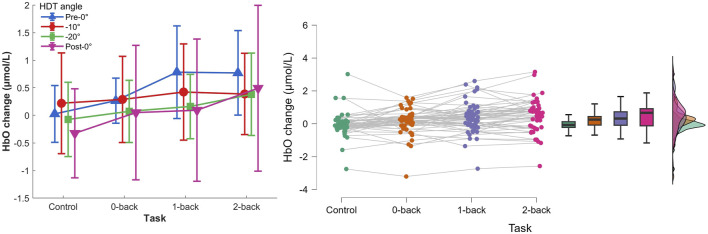
Left) HbO concentration changes per HDT angle and task conditions. Vertical bars represent standard deviation. Right) Raincloud plot of HbO concentration changes per task condition.

The two-way repeated measures ANOVA (Control, 0-back, 1-back, and 2-back × Pre-0°, −10°, −20°, and Post-0°) showed a main effect of task on HbO concentration changes, Figure 7, $F\left(3,36\right)=7.441,p=.013,\eta_{p}^{2}=.383$ . There was no HDT effect, $F\left(3,36\right)=0.655,p=.481,\eta_{p}^{2}=.052$ , nor interaction of both factors, $F\left(9,108\right)=1.571,p=.133,\eta_{p}^{2}=.116$ .

The *post hoc* analyses showed that HbO concentration changes were significantly higher during 1-back and 2-back tasks ( p=.012 and p<.001 respectively), and that HbO changes during 2-back were significantly higher compared with 0-back (p=.038) .

#### 3.2.2 HbR concentration changes

**FIGURE 8:**
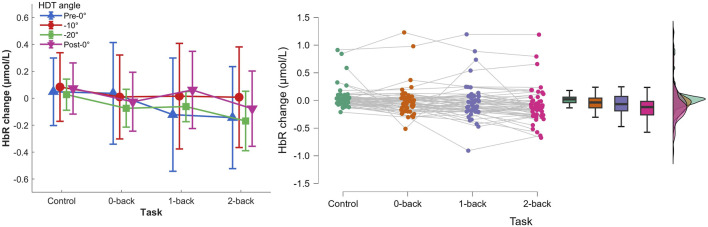
Left) HbR concentration changes per HDT angle and task conditions. Vertical bars represent standard deviation. Right) Raincloud plot of HbR concentration changes per task condition.

The two-way repeated measures ANOVA (0-back, 1-back, and 2-back × Pre-0°, −10°, −20°, and Post-0°) showed a main effect of task, Figure 8, $F\left(3,36\right)=8.018,p<.001,\eta_{p}^{2}=.401$ . No effect of HDT angle, $F\left(3,36\right)=0.876,p=.408,\eta_{p}^{2}=.068$ , nor interaction, $F\left(9,108\right)=1.551,p=.139,\eta_{p}^{2}=.114$ , were found. Post hoc analyses indicated a significantly lower HbR concentration change during 2-back compared to the reaction time task (p<.001) .

## 4 Discussion and conclusion

Microgravity, along with its simulations like head-down tilt, leads to cephalic fluid shift, potentially influencing the assessment of cerebral oxygenation changes. We investigated the effects of simulated microgravity on cerebral oxygenation measures using functional near-infrared spectroscopy (fNIRS) during a cognitive task. The study employed head-down tilt at various inclination levels and the Toulouse N-back Task with different difficulty levels. To record brain activity, we used a simple two-channel portable fNIRS sensor and a basic data processing pipeline, considering future applications for cognitive load monitoring in spaceflight conditions. The emphasis was on simplicity in hardware and data processing for optimal functionality. The objectives of the study were to demonstrate that the cognitive load induced by different difficulty levels yielded significantly different signal amplitudes (HbO and HbR concentration changes), and to quantify the impact of simulated microgravity on these amplitudes.

First, the behavioral results showed that the different levels of difficulty of the Toulouse N-back Task efficiently generated different cognitive loads: with increasing n in n-back, the accuracy decreased, and reaction times and perceived difficulty increased. We also administered a control task where the participants were required only to press a button upon the stimulus display to verify that the reactions were not affected by the tilt level. Taking into account this task, we found faster responses during post-0° compared to pre-0°, likely attributable to a training effect. If we considered only n-back levels (0- vs. 1- vs. 2-back), the tilt angles did not affect the response times. The subjective results of the NASA-TLX questionnaire showed that participants scored the −20° position higher for all dimensions compared to the pre-0° level. However, the scores decreased for the last post-0° level indicating that the previously observed increase was due to the inclination angle and not the time-on-task and fatigue.

Second, the results showed significant variations in oxygenated (HbO) and deoxygenated hemoglobin (HbR) levels as a function of the cognitive load. The HbO increase accompanied by the HbR decrease, in line with the literature, indicated a higher cerebral activity with higher task difficulty. The results were not affected by the head-down tilt angles. It suggests that the simple two-channel fNIRS equipment and simple data processing pipeline can be used for monitoring cognitive load without being impacted by short-term microgravity conditions. It suggests that the previous results on fNIRS measures of cognitive load obtained in normogravity conditions can be applied during spaceflight without specific correction for the fluid shift.

An experiment of longer duration could help strengthen our results and confirm that long space missions would be fully compatible with fNIRS measurements. Also, while the results of this study are promising, the fNIRS signal can present numerous artifacts induced by sensor displacement, facial grimace, or sweating. It forced us to exclude five participants from the analyses as both channels were excessively noisy and consider only one channel for analyses for seven more participants. Hence, a more sophisticated system with an increased number of channels can add redundancy and improve the chances of obtaining a clear signal. These additional channels could be placed near the locations used in this study (above the dorsolateral prefrontal cortex), but could also be added to other parts of the central executive network, a broader network activated by effortful tasks, of which the prefrontal cortex is a part. For example, a study could target the parietal cortex, another critical region of the central executive network (Causse et al., 2022). However, as stated previously, a lower number of fNIRS channels increases the likelihood of the system being accepted, as it is less intrusive.

In summary, the findings demonstrated that increasing difficulty levels in the cognitive task resulted in reduced accuracy, prolonged response times, and increased perceived difficulty scores. Task performance remained unaffected by the inclination levels. Additionally, the results revealed an increase in oxygenated hemoglobin (HbO) and a decrease in deoxygenated hemoglobin (HbR) concentrations with task difficulty, and these trends were not influenced by the head-down tilt angles. These encouraging results suggest that fNIRS measures can serve as a reliable indicator of cognitive load in microgravity conditions without the need for correction due to cephalic fluid shift.

## Data Availability

The raw data supporting the conclusions of this article will be made available by the authors, without undue reservation.
